# Production of recombinant human G protein-coupled estrogen receptor (GPER) and establishment of a ligand binding assay using graphene quantum dots (GQDs)

**DOI:** 10.1371/journal.pone.0332765

**Published:** 2025-09-19

**Authors:** Shakhawat Hossain, Md. Forhad Hossain, Md. Sohanur Rahman Sohan, Yuki Omori, Mohammad Tohidul Amin, Toshinobu Tokumoto

**Affiliations:** Department of Bioscience, Graduate School of Science and Technology, National University Corporation Shizuoka University, Shizuoka, Japan; Nanjing Normal University, CHINA

## Abstract

G protein-coupled estrogen receptor (GPER) is a member of the GPCR family and a key mediator of the rapid, nongenomic actions of estrogens as membrane estrogen receptors. In this study, we established a procedure for the expression and purification of recombinant human membrane estrogen receptor (hGPER) protein via the expression system using the methylotrophic yeast *Pichia pastoris*. By optimizing codon usage, we successfully expressed hGPER at a level that can be purified by column chromatography. The recombinant protein was purified via three chromatography steps. Purified hGPER showed specific estrogen-binding activity (Kd = 9.9 nM and Bmax = 1.76 nM) in a radiolabeled steroid-binding assay. We subsequently established a homogeneous assay for hGPER ligands by conjugating semiconductor nanoparticles known as graphene quantum dots (GQDs) to hGPER. GQDs coupled with hGPER (GQD-hGPER) caused a decrease in fluorescence at 520 nm from E2-BSA-FITC, which was activated by 370 nm light upon the addition of free estradiol to the reaction mixture. Fluorescence was decreased by the administration of hGPER ligands but not by steroids that do not interact with hGPER. Thus, we successfully established a ligand-binding assay for hGPER that is suitable for screening potential compounds. hGPER is a promising candidate for drug discovery for nongenomic estrogen-stimulating effects. The homogeneous assay established in this study will be usable for that purpose.

## Introduction

The nongenomic effects of steroid hormones are a topic of high interest in drug development for a variety of diseases [1]. Although estrogen is best known for its role in the female reproductive system, it also plays a role in cholesterol regulation, bone health protection for both men and women, and a variety of other physiological functions [2–4].

Estrogen signaling through the nuclear estrogen receptor (ER) has been extensively studied and well established [5]. In addition to the membrane progesterone receptor (mPR), a membrane receptor for estrogen was sought after the mPR was identified [6]. One of the orphan 7 trans-membrane (7TM) receptors, GPR30, was eventually identified as a membrane estrogen receptor (mER) [7,8]. Estrogen signaling through the 7TM receptor provides a plausible unifying mechanism for estrogen’s dual actions of activating second messengers and lipid/protein kinases [9]. GPR30 has been shown to be involved in rapid estrogen-dependent G protein signaling and estrogen-specific binding [10]. GPR30 is expressed in a variety of tissues and is thought to transmit estrogenic effects differently in each tissue [11–13]. IUPHAR (International Union of Basic and Clinical Pharmacology) renamed it the G protein-coupled estrogen receptor GPER1 in 2008 [14]. Thus, mER is now known as GPER [15].

Shortly after the initial discovery of GPER, a link between GPER and cancer was shown. Estrogen was shown to be involved in the development of breast and uterine cancers via GPER [16,17]. Involvement in inflammatory signaling in breast cancer and adenocarcinoma has also been suggested [18]. Recently, there have been reports of its involvement in pancreatic ductal adenocarcinoma (pancreatic ductal adenocarcinoma) [19] and other diseases. Estrogen has been shown to act via GPER in the regulation of blood pressure [20], and this activity has been genetically validated [21]. Involvement in ischemic stroke and asymptomatic myocardial ischemia has also been reported [22]. Furthermore, the GPER pathway has been shown to be involved in aldosterone-mediated hypertension and aldosterone synthesis [23,24], and analyses of GPER knockout mice have demonstrated that cardiac contractile (systolic myocardial function) and diastolic (diastolic myocardial function) functions are improved in GPER knockout mice [25], suggesting that the GPER pathway is extensively involved in the regulation of the vascular system.

Since it is now clear that some of the broad hormonal effects of estrogen are mediated by the GPER pathway, research is under way to apply GPER ligands as drugs. Animal studies have demonstrated the effects of G-1, an agonist of GPER, on multiple sclerosis (MS) [26,27], and antagonists of GPER have been shown to act as down regulators of Nox1 [28]. Recently, G-1 has also received attention for its obesity-suppressing effects [29].

Thus, the identification of specific GPER ligands may lead to the identification of new pharmaceuticals. However, the purification of receptor proteins, particularly solubilization and fractionation, is a difficult task. Although the GPER protein is a therapeutic target, no large-scale production and purification of active human GPER protein (hGPER) has been reported to date. Therefore, we aimed to express and purify the active hGPER protein.

In this study, we established procedures for the expression and purification of hGPER and established a homogeneous assay system for determining the compounds that interact with GPER. Previously, we identified the first mPR antagonist from the sea alga *Padina* via the GQD-hmPR assay [30]. The homogeneous assay for GPER provides a new way to identify novel ligands related to the nongenomic actions of estrogen.

## Results

### Recombinant human GPER protein expression

**Fig 1:**
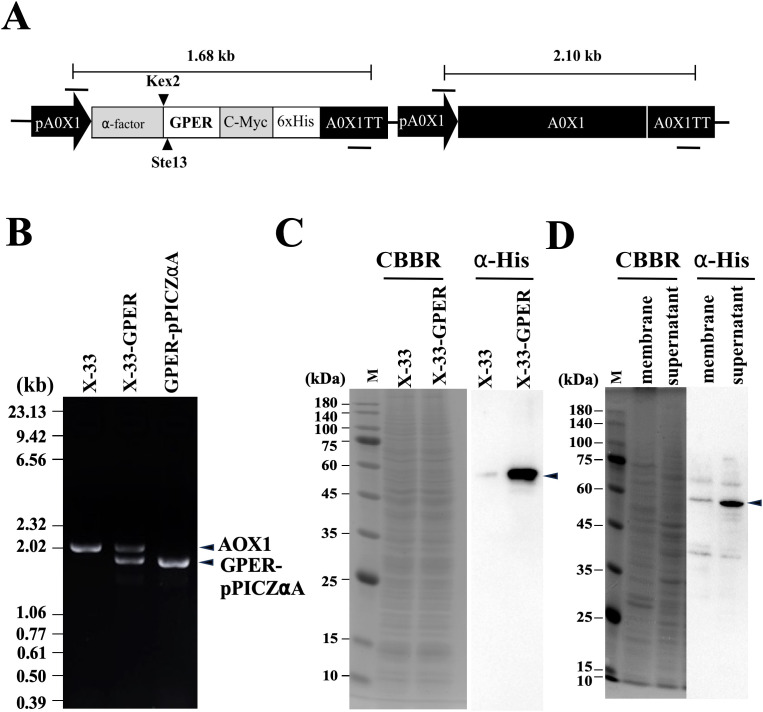
Expression of human GPER in *Pichia pastoris.* (A) Schematic representation of the hGPER expression cassette that was inserted into yeast cells to produce the hGPER protein. The fusion peptide consists of hGPER, an α-factor signal sequence, a C-terminal 6x His tag, and a c-Myc epitope. These are controlled by the methanol-inducible AOX1 promoter (pAOX1) and AOX1 transcription termination region (AOX1 TT) (1.68 kb). The black bars above and below the cassette indicate the 5′AOX1 (F primer) and 3′AOX1 (R primer) primer binding sites, respectively. The AOX1 gene of the yeast cells remained within the expression cassette (2.1 kb). (B) Gene insertion was verified by PCR. DNA fragments were amplified using genomic DNA from either untransformed (X-33) or hGPER-transformed (X-33-GPER) yeast cells, or transformed vector (GPER-pPICZαA) DNA as templates. (C) Expression of hGPER was determined by Western blot analysis. After protein expression was induced in culture with methanol, samples were taken at 6 hours. A protein band of 54 kDa was reacted with an anti-His-tag antibody in the extract prepared from hGPER-transformed cells (X-33-GPER). (D) The distribution of hGPER was determined by Western blot analysis. The precipitate (membrane) and supernatant from the cell extract were separated via centrifugation at 20,000 × g and analyzed via Western blotting. A protein band of 54 kDa for hGPER was detected predominantly in the supernatant.

We constructed a pPICZαA expression vector of hGPER according to the method previously established for mPR and attempted to express it by introducing it into a *Pichia* strain, but the first expression strain prepared with human cDNA presented very low expression levels. Therefore, we decided to optimize the codon usage of hGPER for *Pichia* strains [31]. The optimized sequence was designed, and the hGPER coding region was constructed via artificial synthesis (S1 Fig). The construct was inserted into the host yeast genome via homologous recombination. The successful insertion of the cassette, along with its promoter and terminator, which control the transcription of the heterologous GPER gene fusion, into yeast cells was confirmed via PCR using *AOX1* primer sets (Fig 1). The construct was incorporated into an expression vector, and expression was induced again and achieved at levels detectable by CBBR staining. The expression of the fusion protein, which also carried a c-Myc epitope and a histidine tag at its C-terminal end (Fig 1B), was confirmed by Western blot analysis via anti-His tag antibodies. A single band at approximately 54 kDa was detected (Fig 1C). The molecular weight estimated from the amino acid sequence of human mER is 42 kDa. However, as Fig 1A shows, the recombinant mER has an α-factor sequence added to its N-terminus, as well as Myc and His tags added to its C-terminus. This results in a molecular weight of approximately 54 kDa. After we confirmed the expression of our desired protein, cell pellets from a large culture were disrupted via a ball mill and processed to separate the precipitate (membrane fraction) and the supernatant via centrifugation. In contrast to the case of mPR, Western blot analysis confirmed that the supernatant contained a greater amount of protein than did the precipitate (membrane fraction) (Fig 1D). Therefore, we attempted to purify hGPER from this supernatant.

### Purification of hGPER

**Fig 2:**
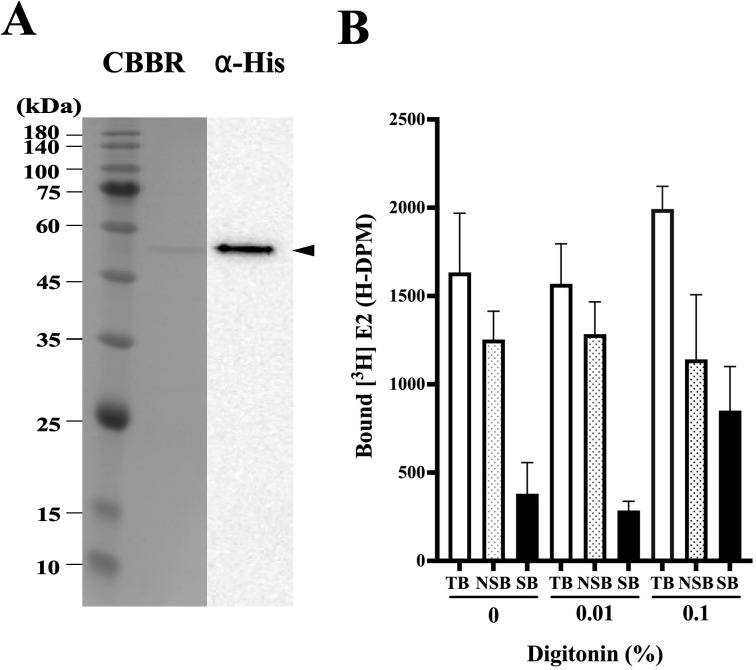
Purification of hGPER and estradiol binding activity of purified protein. (A) Protein bands were detected using CBBR staining or immunostaining with an anti-His tag antibody (α-His). An arrowhead indicates hGPER. (B) The binding activity of hGPER to [2,4,6,7 ^3^H]-estradiol was measured at different concentrations of digitonin (0, 0.01 and 0.1%). The total binding activity (TB) without a competitor and the nonspecific binding activity (NSB) with 1 µM estradiol as a competitor were measured. Specific binding (SB) to soluble hGPER was calculated from TB and NSB.

In several preliminary trials, we established three-step column chromatography for the purification of hGPER. For the first step, we selected Sephacryl S-300 HR gel chromatography resin. The presence of the desired hGPER protein was confirmed in fractions corresponding to the monomer of hGPER by Western blotting analysis (S2 Fig). The fractions were subsequently pooled and separated on a Ni-NTA agarose column. Then, the GPER fractions were further separated on a Cellufine Amino (JNC Corporation, Tokyo, Japan) column. SDS‒PAGE with Coomassie Brilliant Blue (CBB) and immunoblotting (Fig 2A) demonstrated that the recombinant hGPER was successfully purified. Identity of hGPER was confirmed by TOF-MS analysis of purified protein (S3 Fig).

### Specific binding of [^3^H]1,2,6,7-estradiol to purified hGPER

**Fig 3:**
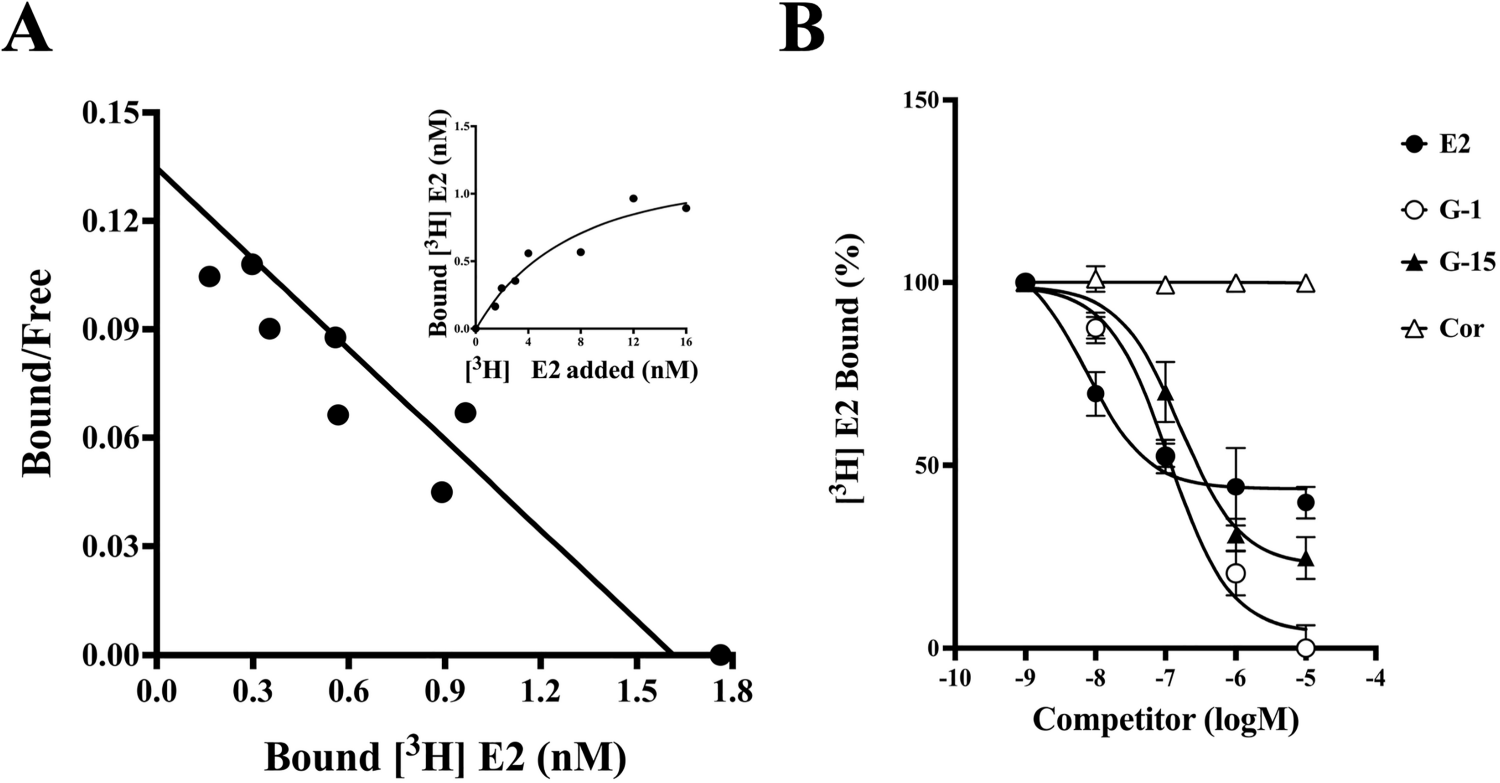
Scatchard plot analysis and ligand specificity of purified hGPER. (A) Saturation curves and Scatchard plots of specific [2,4,6,7 ^3^H]-estradiol binding to hGPER. (B) Competition by steroids and ligands for hGPER. The effects of the steroids estradiol-17β (E2) and cortisol (Cor) and the GPER ligands G-1 and G-15 were assessed in a dose-dependent manner. An assay was performed in triplicate for each compound. Competition for [^3^H]-estradiol binding is expressed as a percentage of the maximum specific G-1 binding.

Digitonin was used to measure the activity to demonstrate the specific binding of [^3^H]1,2,6,7-estradiol to the purified hGPER protein because this glycoside facilitates steroid receptor access [32,33]. A previous study reported that a final concentration of 0.1% digitonin was optimal for facilitating steroid binding [34], as measured by a filter-binding assay. In the case of hGPER, digitonin also had a positive effect on specific binding (Fig 2B). Thus, we added 0.1% digitonin to the reaction mixture for the steroid binding assay. Saturation analysis demonstrated that estradiol binding to hGPER is saturable and has a limited capacity (Bmax = 1.76 nM). Scatchard analysis indicated the presence of a single high-affinity binding site (Kd = 9.88 nM) (Fig 3A). The specificity of binding was also confirmed with the GPER-selective agonist G-1 and the antagonist G-15 (Fig 3B). hGPER specifically bound to E2, G-1 and G-15 but not to cortisol. These results indicated that the purified recombinant hGPER was active and bound specifically to E2. Next, we attempted to establish a homogeneous assay using purified hGPER as hmPR.

### Establishment of a homogeneous assay for GPER ligands

**Fig 4:**
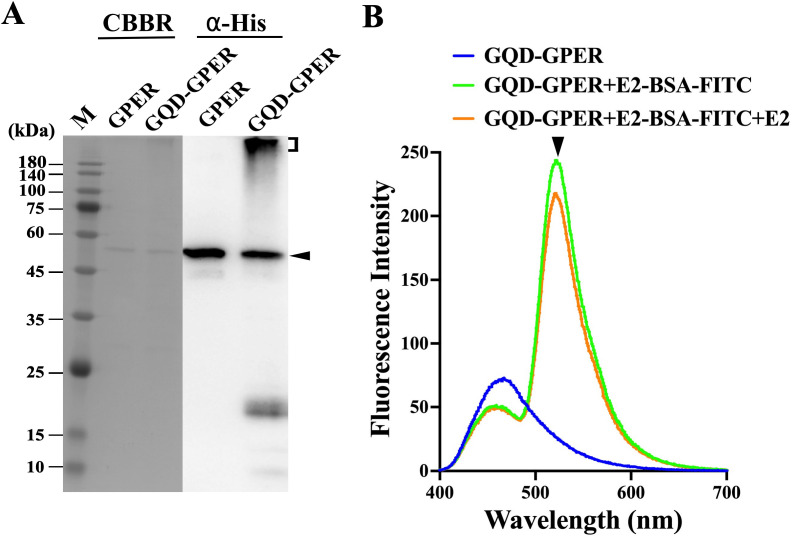
Establishment of the GQD-binding assay. (A) Western blot analysis of hGPER and GQD-hGPER. The protein band of hGPER is indicated by an arrowhead. The bands of GQD-hGPER are indicated in parentheses. (B) The fluorescence scanning pattern of free GQD-hGPER with excitation at 370 nm is indicated by the blue line. The fluorescence scanning pattern of the reaction mixture (GQD-hGPER + E2-BSA-FITC) with (orange line) or without free E2 (green line) is shown. The change in the fluorescence peak at 520 nm caused by the addition of free E2 is indicated by the arrowhead.

Recently, our laboratory established a homogeneous assay utilizing fluorescent nanoparticle graphene quantum dots (GQDs) to investigate the interaction of compounds with human membrane progesterone receptor alpha (hmPRα) [35], goldfish membrane progesterone receptor α (GmPRα) [36], and zebrafish nuclear progesterone receptor (zPgr) [37]. Similarly, we aimed to establish a fluorescent nanoparticle-based screening assay for hGPER. As established previously, we tried to couple the hGPER protein with GQDs. After the coupling of purified recombinant hGPER with GQD, the successful GQD-hGPER conjugate appeared as high-molecular-weight bands (Fig 4A). Next, we attempted to establish a homogeneous binding assay using fluorescently labeled estradiol (E2-BSA-FITC) with GQD-hGPER. After optimization of the reaction conditions, the fluorescence scanning pattern of GQD-hGPER showed a maximum excitation at 370 nm and a maximum emission peak at 470 nm. When E2-BSA-FITC was added to the reaction mixture with GQD-hGPER, the fluorescence scanning pattern presented a double peak, where the first peak at 470 nm corresponded to free GQD-hGPER and the larger peak at 520 nm corresponded to the combined fluorescence of GQD-hGPER and E2-BSA-FITC. The fluorescence intensity at 520 nm decreased upon the addition of free estradiol to the reaction mixture (Fig 4B). This result demonstrates that the addition of free estradiol to the reaction mixture releases the binding between GQD-hGPER and E2-BSA-FITC, resulting in fluorescence via a fluorescence resonance energy transfer (FRET) mechanism. Then, we tried to determine the specificity of this binding assay by checking the competitive binding activity of different steroids and EDCs under these optimized conditions.

**Fig 5:**
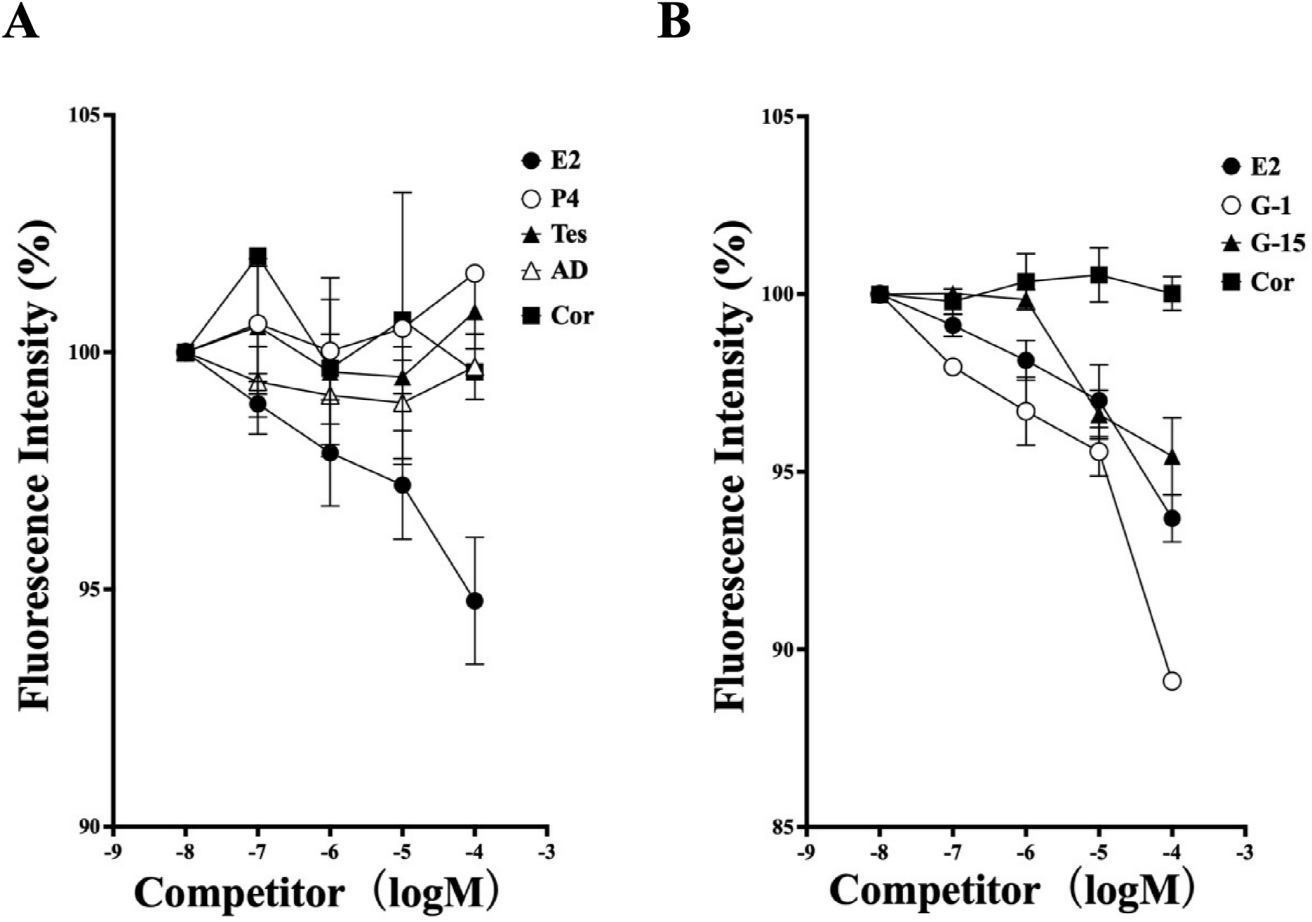
Specificity of the homogenous assay using GQD-hGPER. (A) Competition of the binding of E2-BSA-FITC with GQD-hGPER by steroids. The effects of steroids (estradiol-17β (E2), progesterone (P4), testosterone (T), andorostenedion (AD), and cortisol (Cor)) were assessed in a dose-dependent manner. (B) Competition of binding of E2-BSA-FITC with GQD-hGPER by GPER ligands. The effects of the GPER ligands G-1 and G-15 were compared with those of E2 and Cor. An assay was performed in triplicate for each compound. The average of the three assays is indicated relative to the fluorescence intensity measured with the lowest concentration of compound (10 nM) set to 100 and presented with the standard deviation.

Competitive binding assays revealed that the steroid binding of this receptor was specific for E2 (17β-estradiol). Cortisol, androstenedione, testosterone, and progesterone had no affinity for the receptor at concentrations up to 100 μM (Fig 5A). Furthermore, G-1 and G-15 showed competitive binding activity in the GQD-binding assay and the radiolabeled steroid-binding assay (Fig 5B). We concluded that we had successfully established a nanoparticle-based homogeneous assay for detecting hGPER ligands.

## Discussion

In this study, we successfully purified recombinant hGPER via a 3-step purification process. The expression of hGPER in *P. pastoris* enables codon optimization for the efficient production of large quantities of protein. Although codon optimization was necessary for GPER, the results are consistent with those of previous studies demonstrating the feasibility of expressing and purifying receptors with hormone-binding activity, such as hmPRα and GmPRα [34,38]. However, a relatively large amount of recombinant hGPER was expressed in the soluble fraction. This is in contrast to hmPRα and GmPRα, which were found in the membrane fraction of *Pichia* cells and required detergent solubilization for purification. Thus, hGPER was purified directly via column chromatography without solubilization. The hGPER in the fractions corresponded to the monomeric protein in gel chromatography. Adding 0.01% DDM to the chromatography buffer may support protein solubilization, but the results suggested that hGPER was expressed as a soluble monomer in *Pichia* cells. This special characteristic of hGPER resulted in high purity. The Kd value of purified hGPER was comparable to that of hGPER expressed in cancer cell lines [7]. We were able to express and purify hGPER in its active form.

Notably, we established a ligand binding assay for hGPER using graphene quantum dots (GQDs), similar to recent advances in assay systems for hmPRα and GmPRα [35,36]. This approach allowed us to detect ligand interactions via FRET, leveraging the unique properties of GQDs. GQD-hGPER specifically bound to E2 (17β-estradiol). Specificity was also confirmed by the GPER-selective agonist and antagonist G-1 and G-15 [39,40]. Other steroids, such as P4 (progesterone), cortisol, and testosterone, showed no affinity for the receptor at concentrations up to 1 µM [9].

Attempts to identify GPER ligands via virtual screening were already successful nearly 20 years ago, leading to the discovery of G-1 and G-15 [39,40]. However, we have developed an assay using real recombinant proteins. Using a similar method, we successfully identified a novel ligand for hmPRα from the sea alga *Padina*. We believe that this homogeneous assay is still effective for the discovery of new compounds.

GPER has been found to be expressed in the reproductive organs (ovaries, uterus, and testes), as well as in the kidneys, brain, and spinal cord of vertebrates [7]. This broad distribution suggests that GPER participates in a variety of estrogen-related functions in tissues. A homogeneous ligand binding assay can be applied for screening new compounds from various sources. Recently, we began screening natural compounds from sea algae that interact with hGPER, as we did with hmPRα [30].

## Conclusion

In conclusion, our study successfully purified recombinant hGPER via a combination of size exclusion, affinity chromatography, and ion exchange chromatography. Our novel ligand binding assay, which uses graphene quantum dots (GQDs), demonstrated high sensitivity in detecting ligand interactions via FRET. The GQD-hGPER assay provides a robust platform for screening agonists and antagonists, facilitating the discovery of new selective therapeutics for cancer and other diseases. This approach offers promising insights and potential advancements in the fields of hormone receptor research and drug development.

## Methods

### Materials

Digitonin was purchased from Sigma‒Aldrich Chemicals (St. Louis, MO). E2-BSA-FITC was purchased from Steraloids, Inc. (Newport, R.I.). Dodecyl β-D-maltoside (DDM) was purchased from Dojindo (Tokyo, Japan). A Sephacryl S-300 high-resolution column was purchased from Pharmacia Fine Chemicals (New Jersey, USA). G-1 was purchased from Angene International Limited (Angene International Limited, Hong Kong). G-15 was purchased from Tocris Bioscience Tocris Bioscience (Bristol, UK). [2,4,6,7 ^3^H]-Estradiol was purchased from Revvity™ (Revvity, Germany).

Other chemicals were purchased from Wako Pure Chemical Industries, Ltd. (Osaka, Japan).

### Construction of the recombinant pPICZαA plasmid

The codon-optimized DNA sequence of hGPER was designed via software according to codon usage in *Pichia*. The production of artificial DNA was performed by an outside company (Eurofins Japan). The DNA template for the human GPER of the pBluescript II KS(+) plasmid was amplified using polymerase (KOD Plus Neo, TOYOBO, Japan) and the primer set of Hs GPER-EcoRI, CGGAATTCATGGATGTGACTTCCCAAGCCCGGGG, and Hs GPER-NotI, ATAAGAATG CGGCCGCCACGGCACTGCTGAACCTCACATCCG. To express the GPER in *P. pastoris*, it was inserted into the expression vector pPICZαA (Invitrogen). The ORF region DNA sequences of the expression vectors were verified via DNA sequencing.

The *P. pastoris* X-33 strain (Invitrogen) was transformed with the hGPER expression construct via electroporation, as previously described in detail [34]. The construct was linearized via PmeI digestion, and 168 μg of the linearized plasmid was used to transform *P. pastoris* cells through electroporation. Electroporation was performed using a Gene Pulser instrument (Bio-Rad) according to established protocols [34].

Plates containing yeast extract–peptone–dextrose (YPD) medium (1% yeast extract, 2% peptone, 2% dextrose, and 2% agar), supplemented with 500 μg/mL Zeocin were selected for the culture of the recombinant colonies. We verified the genomic amalgamation of the hGPER construct via PCR using Ex Tag Polymerase (Takara Bio, Siga, Japan) and the primer set 5′AOX1 (GACTGGTTCCAATTGCAAAGC) and 3′AOX1 (GCAAATGGCATTCTGACATCC) to amplify the sequence between the AOX1 promoter and terminator regions (Fig 1A). Several Zeocin-resistant clones were analyzed to confirm the production of the recombinant protein.

The clones with the highest expression levels were maintained and stored on MD plates containing 1.34% yeast nitrogen base, 4 × 10^−5^% biotin, 2% dextrose, and 1.5% agar at 4 °C [34].

### Expression of hGPER in *P. pastoris*

A single colony was selected and grown in 100 ml of BMGY medium (1% yeast extract, 2% bactopeptone, 1 M potassium phosphate at pH 6.0, 1.34% yeast nitrogen base without amino acids, 4 × 10^−5^% biotin, and 1% glycerol) at 30 °C with shaking at 180 rpm for 21 hours. The culture was then scaled up to 500 mL in a 2-L baffled flask. It was incubated for an additional 16.5 hours under the same conditions and reached an optical density (OD at 600 nm) of 14–15. The cells were washed with 100 ml of BMMY medium (1% yeast extract, 2% bactopeptone, 1 M potassium phosphate at pH 6.0, 1.34% yeast nitrogen base without amino acids, 4 × 10^−5^% biotin, and 0.5% methanol) to prepare for protein induction. The cells were resuspended in 400 mL of BMMY at an OD600 of 15–16. They were then placed in a 2-L baffled flask and incubated at 20 °C for six hours with shaking at 180 rpm to induce hGPER protein expression.

Finally, the cells were harvested by centrifugation at 3000 × g for 5 minutes, frozen with liquid nitrogen, and stored at –80 °C.

### *Pichia* cell disruption and preparation of the soluble fraction

The frozen cell pellets (approximately 60 g), which were harvested from a 6 L culture, were thawed and suspended in 250 mL of ice-cold breaking buffer (50 mM sodium phosphate, 1 mM PMSF, 1 mM EDTA, and 5% glycerol, pH 7.4). The suspension was then refrozen in tubular form in a stainless-steel chamber for cell disruption, utilizing 5 stainless-steel balls. Subsequently, cell disruption was carried out via a Retsch Ball Mill PM 100 (Verder Scientific Co., Ltd., Haan, Germany), with six cycles of shaking interspersed with intervals of chilling using liquid nitrogen. Each shaking cycle was performed at 400 rpm for 3 minutes. The resulting broken cells, resembling a white powder, were collected into centrifuge tubes. Unbroken cells and debris were separated from the membrane-containing fractions via low-speed centrifugation (1000 × g, 4 °C, 7 min). Following supernatant collection, the pellet was resuspended in 60 ml of ice-cold breaking buffer for an additional round of supernatant collection. The total supernatants from two consecutive rounds were combined, and the soluble fraction and membrane fractions were separated by centrifugation at 20,000 × g and 4 °C for 20 minutes. The resulting supernatant was poured into a bag with a pore size of 3.5 kDa. The bag, coated with sucrose, facilitated buffer absorption and concentration through osmosis. The concentrated supernatant was subsequently dialyzed for 24 hours in a 3.5 kDa dialysis bag, and the dialysate was replaced every 8 hours with distilled deionized water (DDW). All steps were carried out at 4 °C.

### Purification

The concentrated supernatant containing hGPER protein was applied to a Sephacryl S-300 gel column (φ2.6 × 56.5 cm, 300 ml). Each run involved 10 ml of protein from 50 ml of concentrated supernatant. Elution was performed via CA buffer (50 mM Tris-HCl and 0.15 M NaCl buffer, pH 8.0, containing 0.01% DDM and 1 mM PMSF), and the eluted fractions were collected in 45 tubes (10 ml). The desired protein-containing fractions were identified via Western blotting analysis (using anti-His-tag antibodies) and Coomassie Brilliant Blue (CBB) staining. The identified fractions were subsequently subjected to a Ni-NTA agarose column (80 ml resin) (QIAGEN, Gaithersburg, MD, USA) with dimensions of φ 4.5 × 5.0 cm, which was preequilibrated with lysis buffer (50 mM NaH_2_PO_4_, 300 mM NaCl, 40 mM imidazole, pH 6.0) containing 1 mM PMSF and 0.01% DDM. After sample application, the column was washed with a 5-fold column volume of lysis buffer, followed by elution with a 400 mL gradient of 40–400 mM imidazole in the same buffer (40 mM imidazole and 400 mM imidazole). Finally, the column was washed with 100 ml of elution buffer, which contained 400 mM imidazole. The fractions containing hGPER were confirmed by CBB staining and Western blot analysis via the use of anti-His-tag antibodies. The collected fractions were diluted 4.5 times with DDW. The samples were applied to a 10 mL Cellufine Amino (JNC Corporation, Tokyo, Japan) column (φ 1.6 × 10 cm) equilibrated with CA buffer (50 mM Tris-HCl buffer, pH 8.0, containing 0.01% DDM and 1 mM PMSF). The column was subsequently washed with 80 mL of the same buffer (50 mM Tris-HCl buffer, pH 8.0) and eluted with a 50 mL gradient of 0–0.5 M NaCl in CA buffer. All the purification procedures were conducted at 4 °C.

### SDS‒PAGE and Western blot analysis

Proteins were separated via SDS‒polyacrylamide gel electrophoresis (SDS‒PAGE) via a 12% polyacrylamide gel under denaturing conditions following the Laemmli method [41]. After SDS‒PAGE, the separated proteins were subsequently transferred onto Immobilon membranes (Millipore, Billerica, MA) via a WSE-4115 semidry transfer system (ATTO, Tokyo, Japan). The membranes were blocked for 1–2 hours at room temperature with 5% nonfat powdered milk in 20 mM Tris-buffered saline (TBS), containing 0.1% Tween-20 (TTBS). After incubation with anti-His-tag primary antibodies (1,000-fold dilution in TBS buffer), the membranes were washed with TTBS three times at 5-minute intervals. The membranes were subsequently incubated with goat-anti-mouse secondary antibodies (2,000-fold dilution in TBS buffer), followed by another three washes with TTBS at 5-minute intervals. The target protein was visualized using an enhanced chemiluminescence reaction using an ECL detection kit (PerkinElmer, Waltham, MA). This method is based on a chemiluminescent reaction mediated by a peroxidase-conjugated secondary antibody. The signals were digitized via a CCD camera system (Luminescent Image Analyzer LAS-4000 mini; Fujifilm, Tokyo, Japan).

### Peptide mass fingerprint analysis of purified GPER

The purified recombinant hGPER proteins, which were stained with CBBR in SDS-PAGE gel slices, were trypsinized. The peptides were then recovered using a ZipTip (Millipore) and eluted through a 2-μL solution containing 60% acetonitrile, 0.1% TFA, and 5 mg/mL CHCA (Bruker Daltonics), as previously described for human mPRα [38]. The samples were loaded into a 384-well plate containing a double layer of CHCA, dissolved in acetone, and air-dried. We used a MALDI-TOF/MS Autoflex (Bruker Daltonics, Billerica, MA) to detect the peptide mass spectrum in positive ion mode. The MALDI-TOF-MS spectra obtained were calibrated using a mixture of molecular weight standards (Bruker Daltonics). Using MASCOT software (Matrix Science, London, UK), we analyzed and compared the peptide fingerprint with peptides from human taxonomy via the NCBInr database. The analysis included cysteine modification by carbamidomethylation (C), a trypsin digest with single missed cleavages, and a peptide mass tolerance of ±0.4 Da. The hGPER protein was identified by its peptide fragment molecular weight via probability-based MOWSE scores.

### Radiolabeled ligand binding assays

The plasma membrane pellet was obtained as described in the Membrane Preparation and Solubilization section. It was then resuspended in HEAD buffer (25 mM HEPES, 10 mM NaCl, 1 mM dithiothreitol, and 1 mM EDTA, pH 7.6), which contained 0.1% digitonin. Progestin ligand binding to the hGPER was measured using previously established procedures [42]. For the binding assay of the solubilized samples, 100 μL of 50% Ni-NTA resin was added. GF/B filters were pre-soaked in a wash buffer that did not contain Tween 80.

### Competition assay

One set of tubes contained 1.5nM [2,4,6,7-^3^H]estradiol alone to measure total binding. Another set contained a 100-fold greater concentration of cold progestin competitor to measure nonspecific binding (NSB). After incubating with the membrane fractions for 30 minutes at 4 °C, the reaction was stopped by filtration using Whatman GF/B filters that had been pre-soaked in a wash buffer containing 2.5% Tween 80. The filters were then washed three times with 5 mL of wash buffer (25 mM HEPES, 10 mM NaCl, and 1 mM EDTA at pH 7.4) at 4 °C. The bound radioactivity was subsequently measured via scintillation counting.

### Saturation analyses and Scatchard plots

Various concentrations of [2,4,6,7-^3^H]estradiol (specific activity: 96.6 Ci/mmol) were added to the assay tubes with or without 100-fold molar excess of cold estradiol (nonspecific or total). We conducted linear and nonlinear regression analyses for all receptor binding assays, as well as calculations of Kd and binding capacity (Bmax), via GraphPad Prism for Macintosh (version 4.0c; GraphPad Software, San Diego, CA). The results are shown as Scatchard plots.

### Preparation of GQDs

A 100 mL round-bottom flask containing 2 g of citric acid was heated to 200 °C in a dry oven as part of a standard GQD preparation process. After 5 minutes, the citric acid liquefied and turned a yellowish color. After 20 minutes of heating, an orange color appeared, suggesting the formation of GQDs. In addition, various reaction times ranging from 20 to 45 minutes were investigated, and a reaction time of 25 minutes was selected in this study to produce GQDs with a high quantum yield (QY). To prevent the development of graphene oxides, extra heating time was rigorously avoided. The orange liquid that contained GQDs was then collected and vigorously stirred before being put dropwise into 100 mL of a 10 g/L sodium hydroxide (NaOH) solution. After neutralization to pH 7.0 with NaOH, an aqueous solution of GQDs was obtained and preserved at 4 °C for further use [43].

### Preparation of GQD-coupled hGPER

The amine group/N-terminus of hGPER and the carboxylic acid group of the GQDs were coupled to create GQD-labeled hGPER (GQD-hGPER). This was accomplished at ambient temperature and pH 6.0 via the usual N-ethyl-N’-(3-(dimethyl aminopropyl) carbodiimide (EDC)/N-hydroxy succinimide (NHS) reaction [44]. Briefly, EDC and NHS were added to the GQD solution under vigorous stirring (on a small scale, 3 mg of EDC and 4 mg of NHS were added to 10 mL of GQD solution; on a large scale, 4.5 mg of EDC and 6 mg of NHS were added to 15 mL of GQD solution) [35]. After that, hGPER was added (the protein concentration was 0.312 mg/mL hGPER). The mixture was stirred vigorously for two hours at room temperature. After removing the unreacted compounds, the final solution was dialyzed for 24 hours in a 3.5 kDa dialysis bag, and the dialysate was replaced every 8 hours with distilled deionized water (DDW). Finally, half of the prepared GQD-hGPER samples were stored directly at −30 °C, while the other half were subjected to lyophilization before storage.

### Binding assay utilizing hGPER-GQDs and E2-BSA-FITC

The reaction mixture included 50 μl of phosphate buffer (pH 7.4), 36 μl of DDW, 10 μl of hGPER-GQDs (hGPER-GQDs with an hGPER concentration of 15 µg/mL), 2 μl of E2-BSA-FITC (E2-BSA-FITC concentration of 40 µg/mL) and 2 μl of the test compound for a total volume of 100 μl per well. For these experiments, we used 96-well plates. The prepared reaction mixture was incubated in the dark for 2 hours at room temperature. After incubation, the fluorescence intensity of each well was measured via a Varioskan™ LUX fluorescence microplate reader from Thermo Scientific (Waltham, USA). The measurements were taken at an excitation wavelength of 370 nm and an emission wavelength of 520 nm.

## Supporting information

S1 Fig Alignment of cDNA sequence of hGPER and optimized DNA sequence of hGPER. (DOCX)

S2 Fig Purification of hGPER protein using Sephacryl S-300 gel filtration, Ni-NTA affinity, and amino cellulose chromatography. (DOCX)

S3 Fig MALDI-TOF mass spectrometric analysis of purified recombinant hGPER. (DOCX)

S1 Table Low data for Figs 1–5.
(XLSX)

S1 File Full uncropped gel and blot images. (DOCX)
