# Supplementary material for: Production of recombinant human G protein-coupled estrogen receptor (GPER) and establishment of a ligand binding assay using graphene quantum dots (GQDs)
Source: PLoS One. 2025 Sep 19;20(9):e0332765. doi: 10.1371/journal.pone.0332765 (PMC12448983; doi:10.1371/journal.pone.0332765)
Supplement: S1 Fig — (DOCX) [file pone.0332765.s001.docx]

**S1 Fig.**

Alignment of cDNA sequence of hGPER and optimized DNA sequence of hGPER. Common sequence of cDNA and optimized sequences are boxed.
