# Supplementary material for: Production of recombinant human G protein-coupled estrogen receptor (GPER) and establishment of a ligand binding assay using graphene quantum dots (GQDs)
Source: PLoS One. 2025 Sep 19;20(9):e0332765. doi: 10.1371/journal.pone.0332765 (PMC12448983; doi:10.1371/journal.pone.0332765)
Supplement: S2 Fig — (DOCX) [file pone.0332765.s002.docx]

**S2 Fig.**


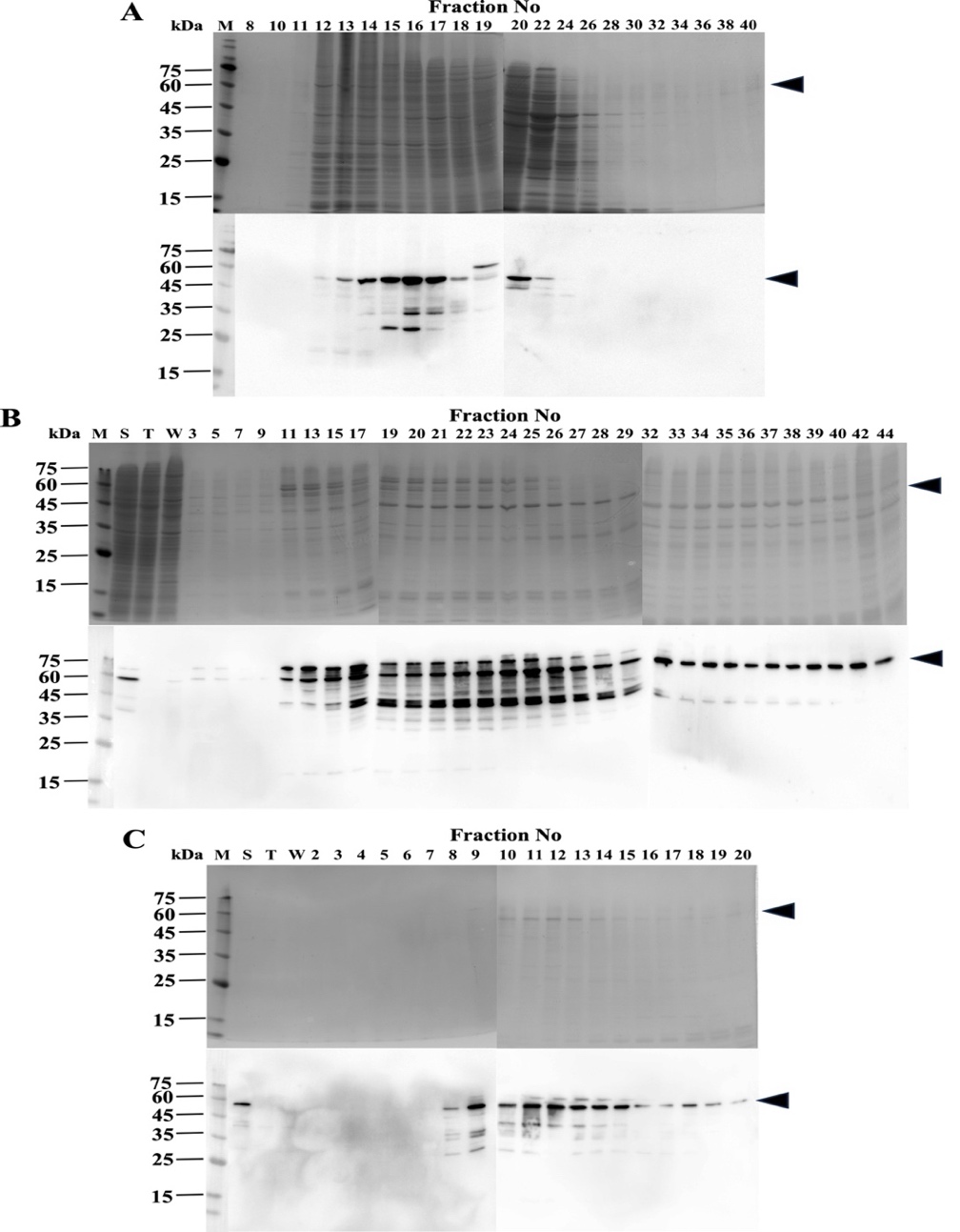


**Purification of hGPER protein using Sephacryl S-300 gel filtration, Ni-NTA affinity, and amino cellulose chromatography**. (A) SDS-PAGE and Western blot analysis of Sephacryl S-300 gel filtration chromatography fractions 14 to 17, which were selected for subsequent purification. (B) SDS-PAGE and Western blot analysis of Ni-NTA affinity chromatography fractions 32 to 42, which were used for further purification. (C) SDS-PAGE and Western blot analysis of amino cellulose chromatography fractions 11 to 13, representing the final purified hGPER protein. Protein bands were visualized by Coomassie Brilliant Blue R-250 (CBBR) staining or immunodetection using an anti-His tag antibody (α-His). Arrowheads indicate the position of the hGPER protein.
