# Supplementary material for: Production of recombinant human G protein-coupled estrogen receptor (GPER) and establishment of a ligand binding assay using graphene quantum dots (GQDs)
Source: PLoS One. 2025 Sep 19;20(9):e0332765. doi: 10.1371/journal.pone.0332765 (PMC12448983; doi:10.1371/journal.pone.0332765)
Supplement: S3 Fig — (DOCX) [file pone.0332765.s003.docx]

**S3 Fig.**

**
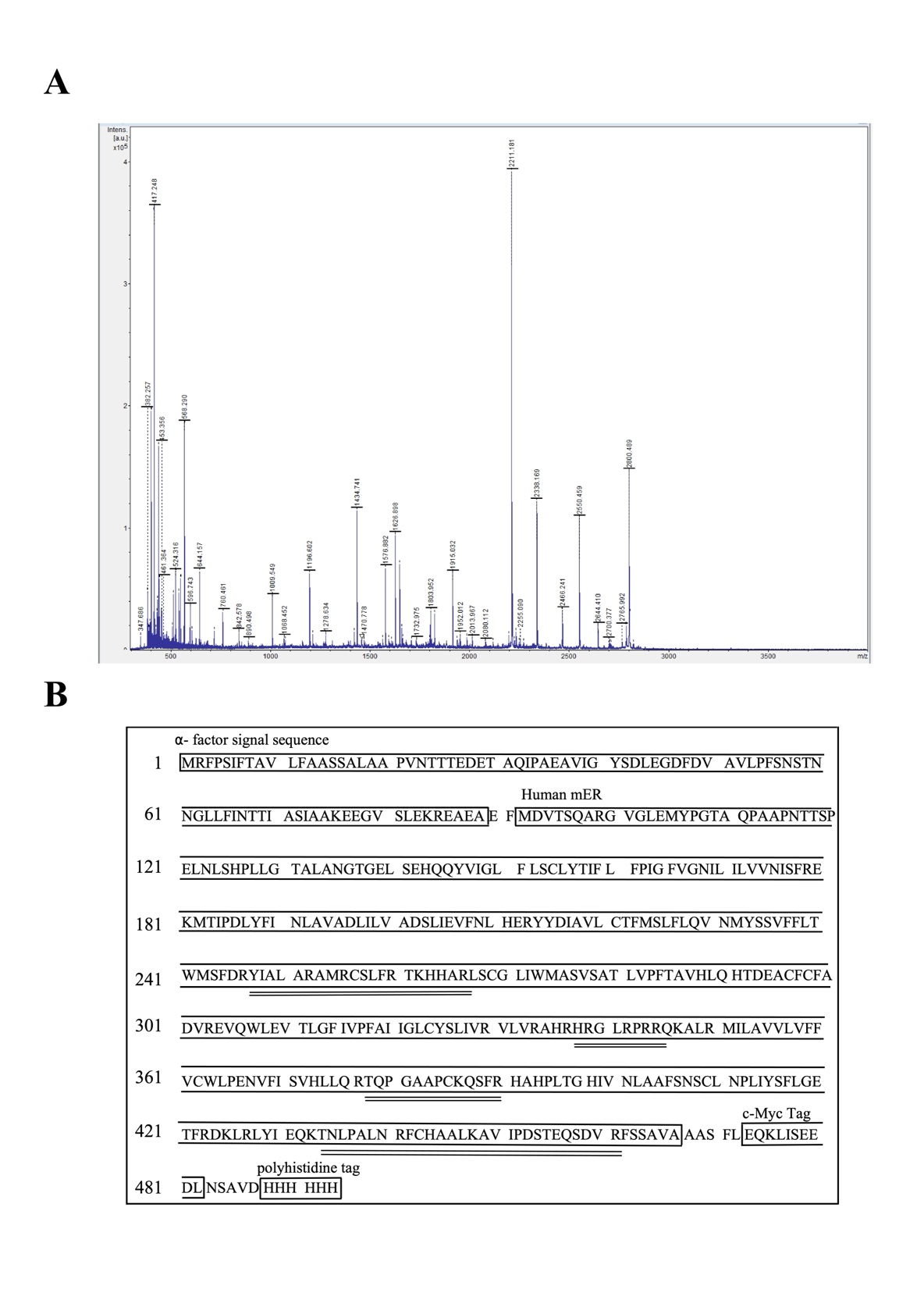
**

**MALDI-TOF mass spectrometric analysis of purified recombinant hGPER.** The amino acid sequence of the recombinant hGPER produced from the construct used in this study is shown. Peptides identified by peptide mass fingerprinting (PMF) analysis are underlined.
