# Supplementary material for: Production of recombinant human G protein-coupled estrogen receptor (GPER) and establishment of a ligand binding assay using graphene quantum dots (GQDs)
Source: PLoS One. 2025 Sep 19;20(9):e0332765. doi: 10.1371/journal.pone.0332765 (PMC12448983; doi:10.1371/journal.pone.0332765)
Supplement: S1 File — (DOCX) [file pone.0332765.s005.docx]

**Full uncropped gel and blot images for Fig. 1C.**

X-33-GPER

X-33

Marker

**
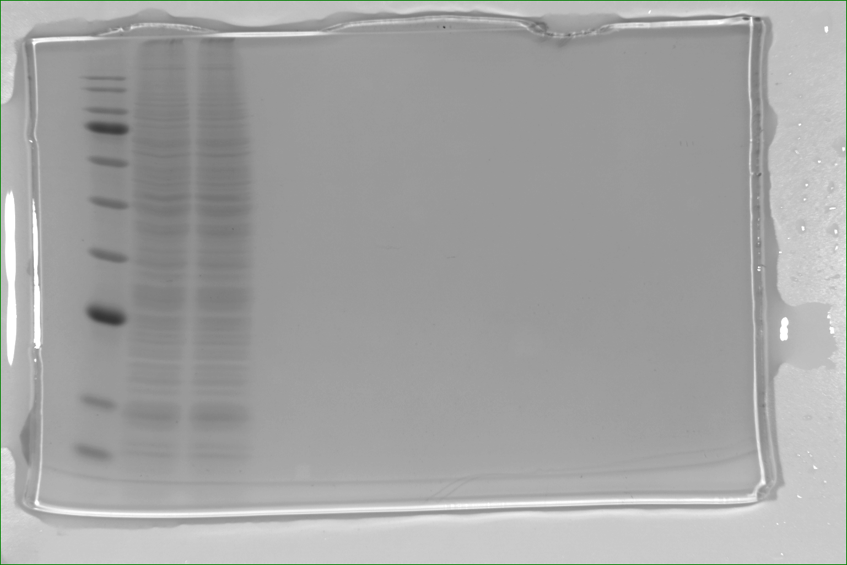
**

Marker

X-33- GPER

X-33

Marker

**
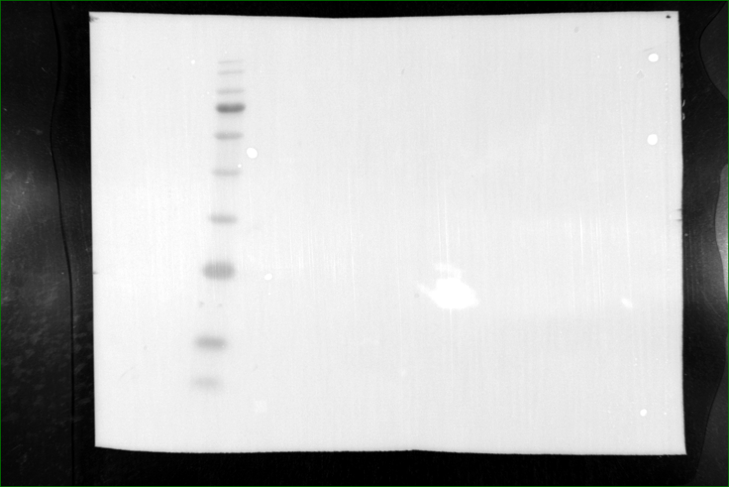

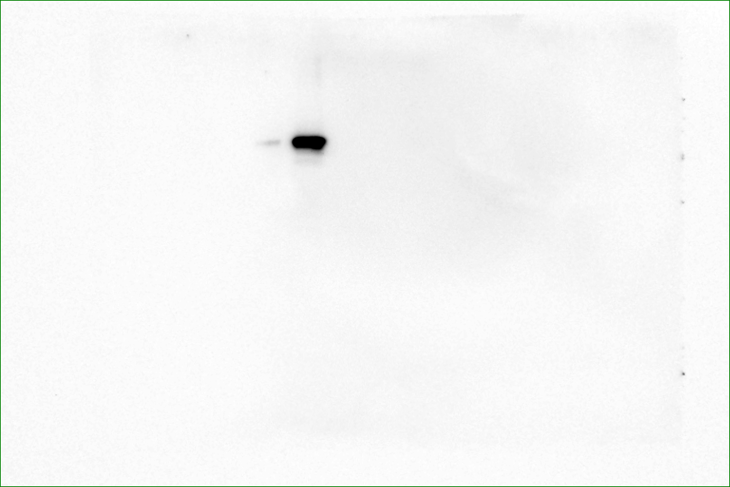
**

These images were obtained following methanol-induced protein expression in culture. The expression of hGPER protein was confirmed by Western blot analysis. A protein band corresponding to hGPER (~54 kDa) is indicated by an arrow.

**Full uncropped gel and blot images for Fig. 1D.**

Supernatant

Membrane

Marker

**
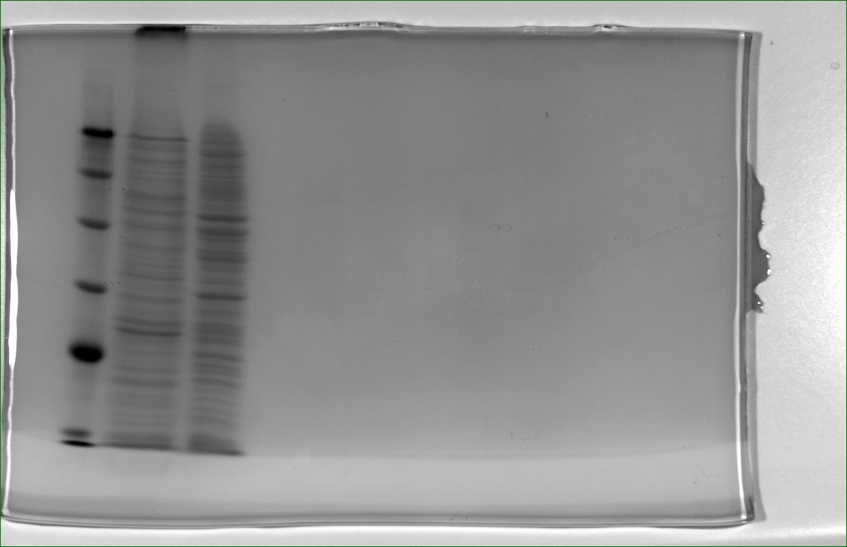
**

Membrane

Supernatant

Marker

**
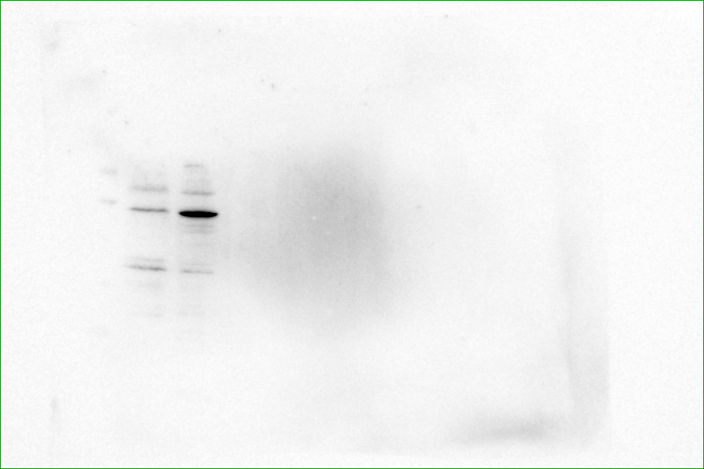

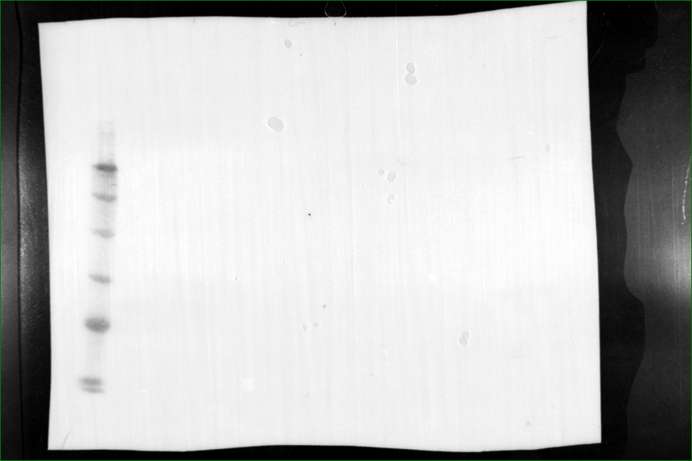
**

The expression of hGPER was detected by Western blot analysis. The precipitate (membrane fraction) and supernatant obtained by centrifuge at 20,000 × g from the cell extract were analyzed. A protein band corresponding to hGPER (~54 kDa) was predominantly detected in the supernatant, as indicated by an arrow.

**Full uncropped Gels and Blots image(s) for Fig. 2**.

Marker

CBBR


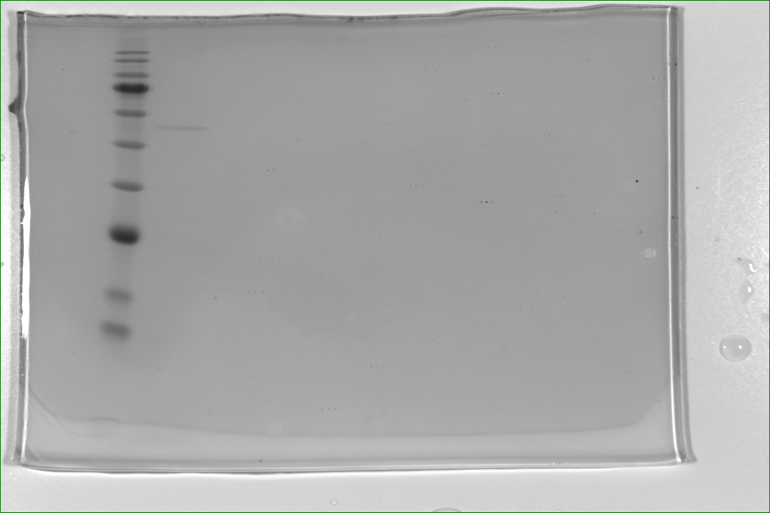


$\alpha$-His


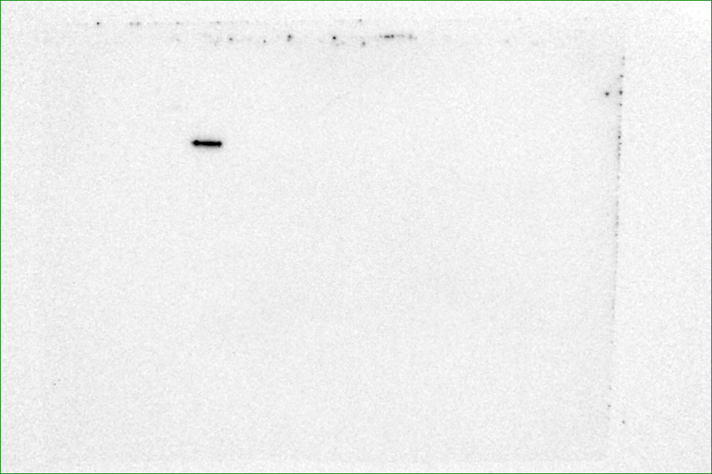

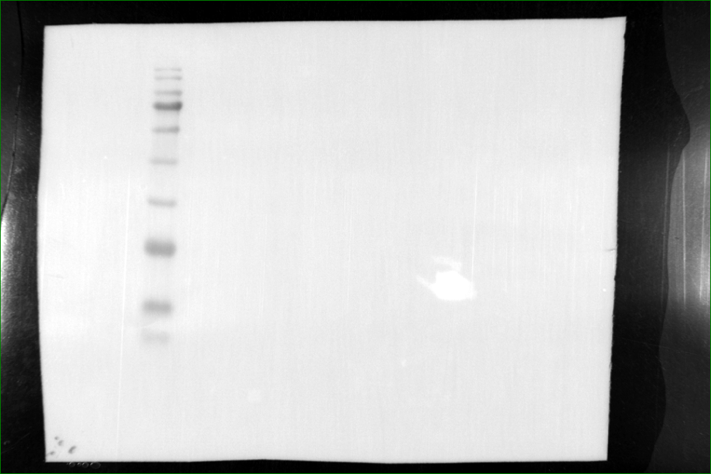


Marker

The hGPER amino cellulose fraction was separated by SDS-polyacrylamide gel electrophoresis (SDS-PAGE) on a 12% polyacrylamide gel and visualized by Coomassie Brilliant Blue R-250 (CBBR) staining and Western blot analysis. Which is indicated by an arrow.

**Full uncropped Gels and Blots image(s) for Fig. 4A.**


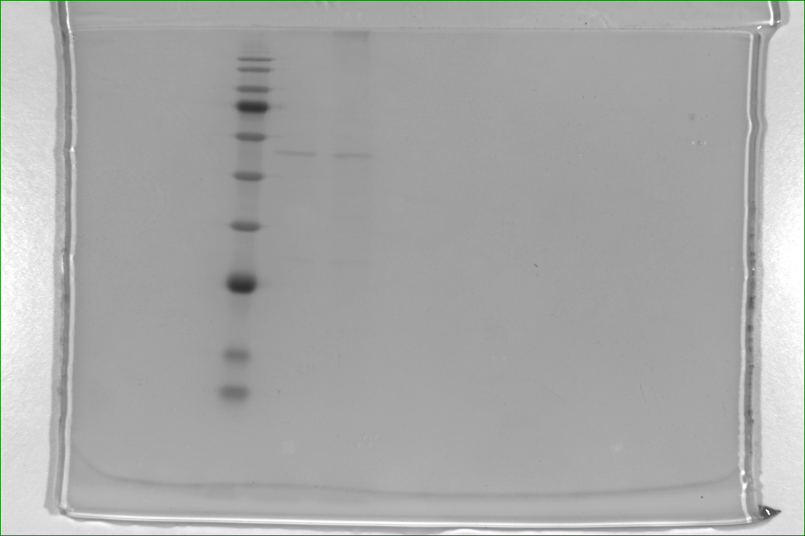


GQD- GPER

GPER

Marker

GQD- GPER

Marker

GPER


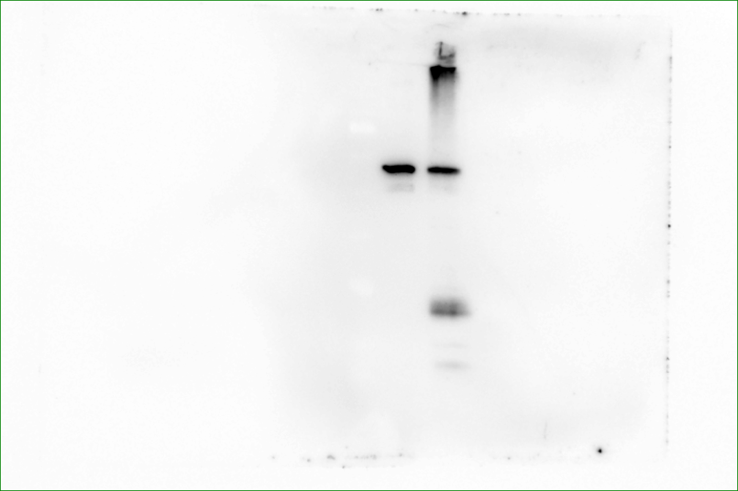

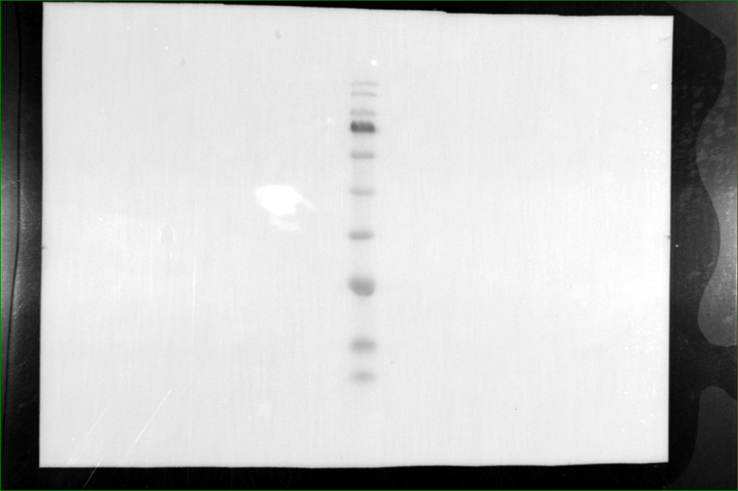


The amino cellulose fractions were conjugated with GQDs (GQD-hGPER). The single band corresponding to the unmodified hGPER protein is indicated by an arrow. The bands corresponding to the GQD-hGPER conjugates are indicated by a bracket in both the Coomassie Brilliant Blue R-250 (CBBR) staining and the Western blot analysis.

**Full uncropped gel and blot image(s) for S2 Fig. A**

**Fraction No**

10

8

11

12

13

15

14

18

16

17

19

M


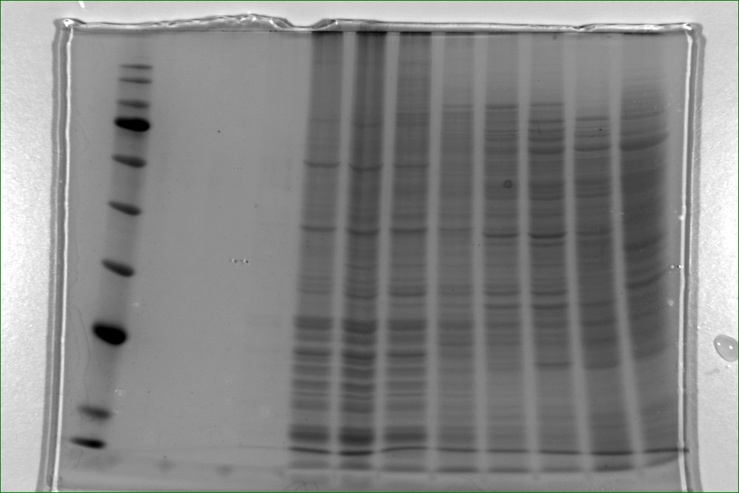


**Fraction No**

10

14

8

11

12

13

15

16

17

18

19

M


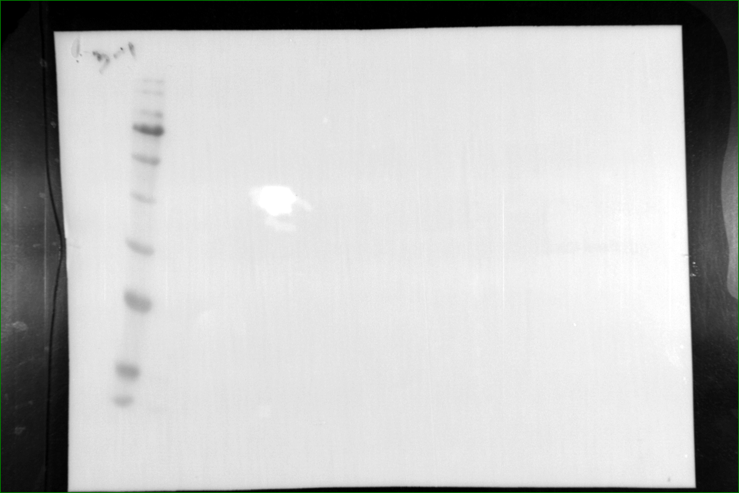

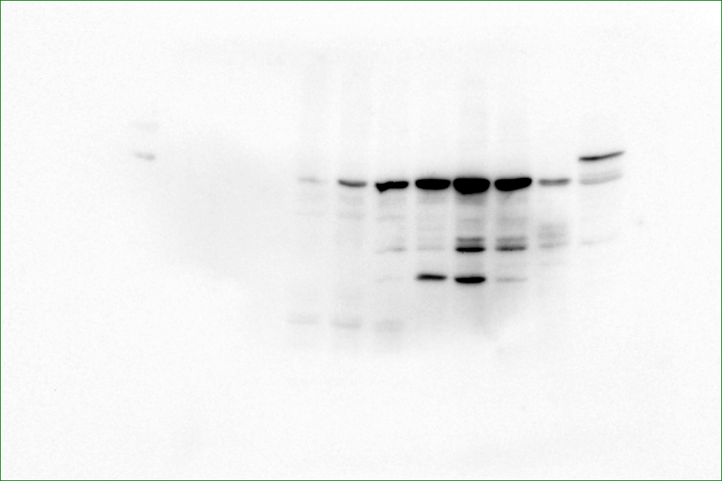


**Fraction No**


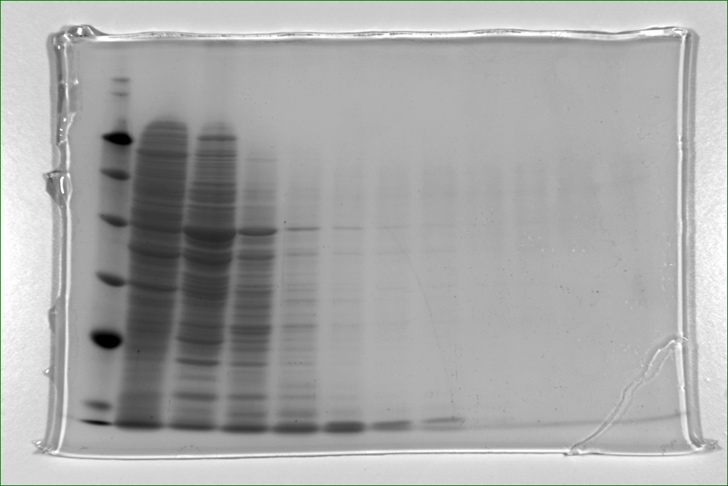


M

**Fraction No**


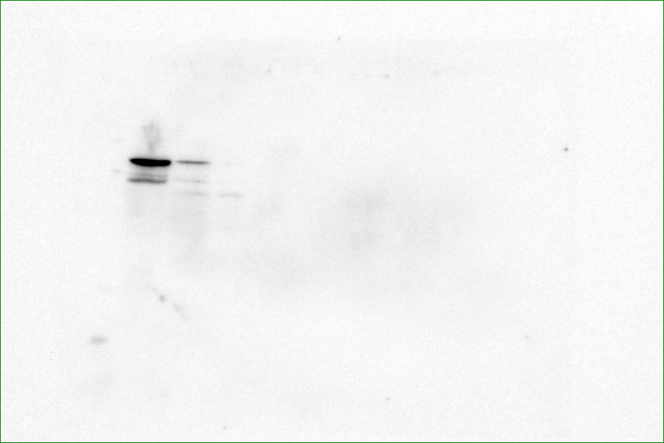

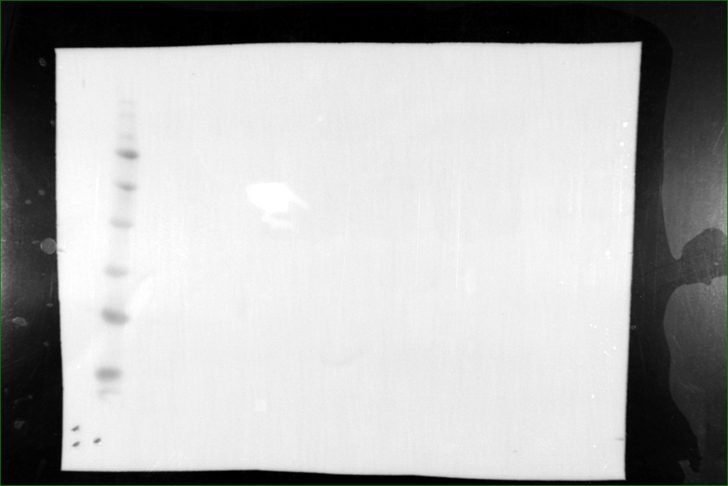


M

**Purification of hGPER protein using Sephacryl S-300 gel filtration chromatography**

**Full uncropped gel and blot image(s) for S2 Fig. B**

**Fraction No**


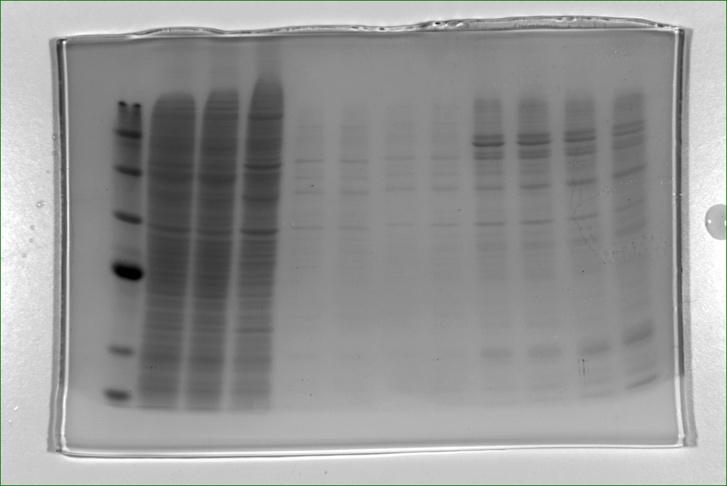


**Fraction No**

M


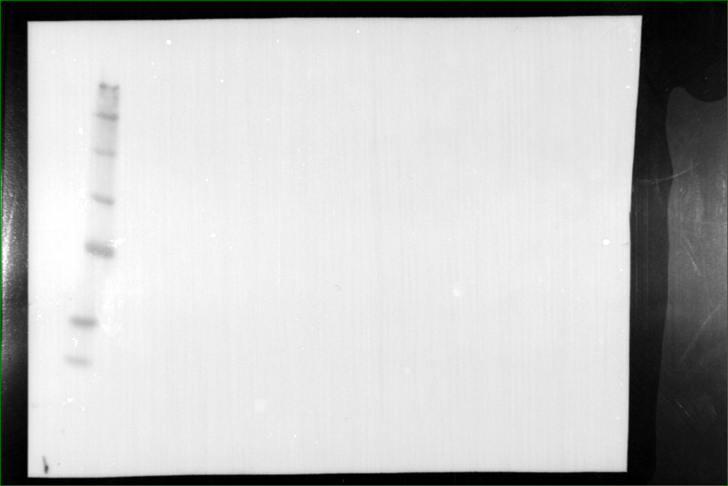


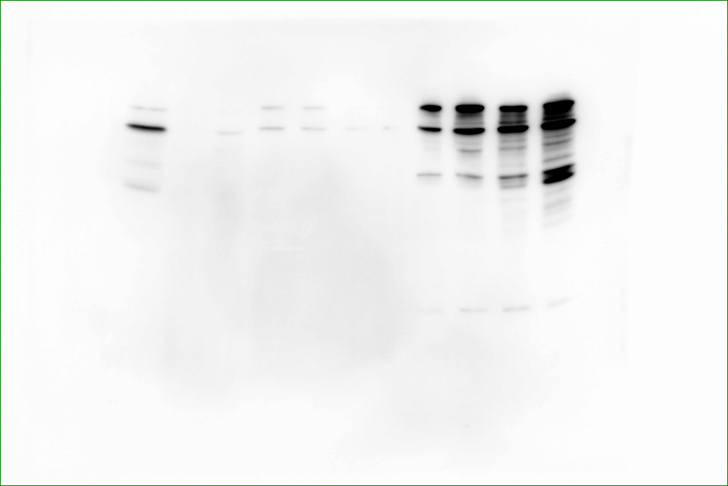


**Fraction No**

M


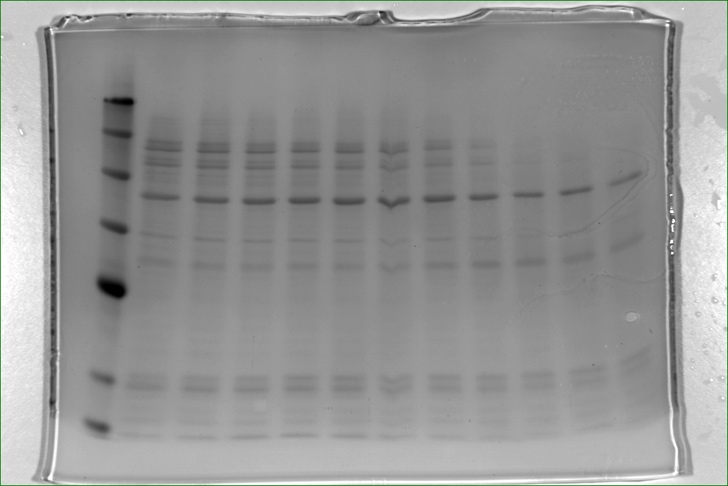


**Fraction No**

M


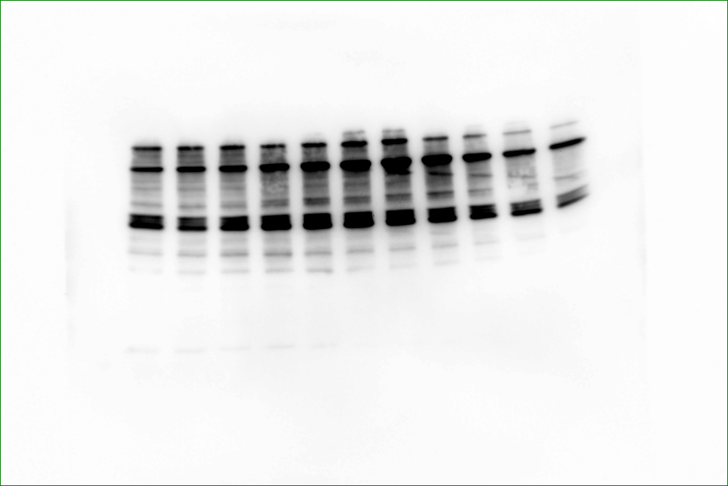

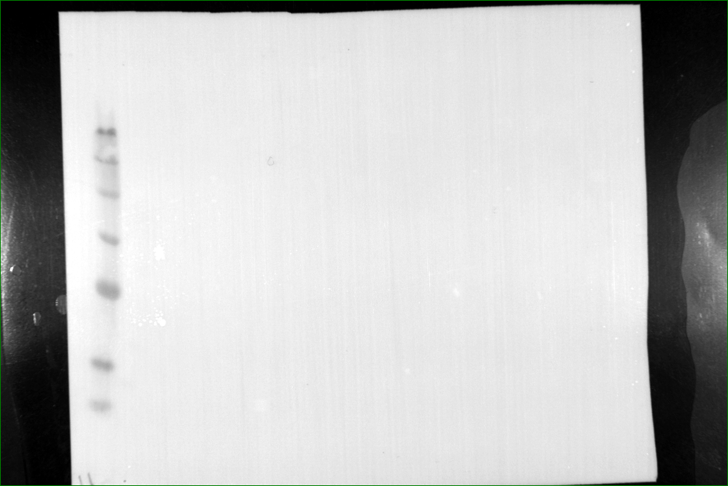


**Fraction No**


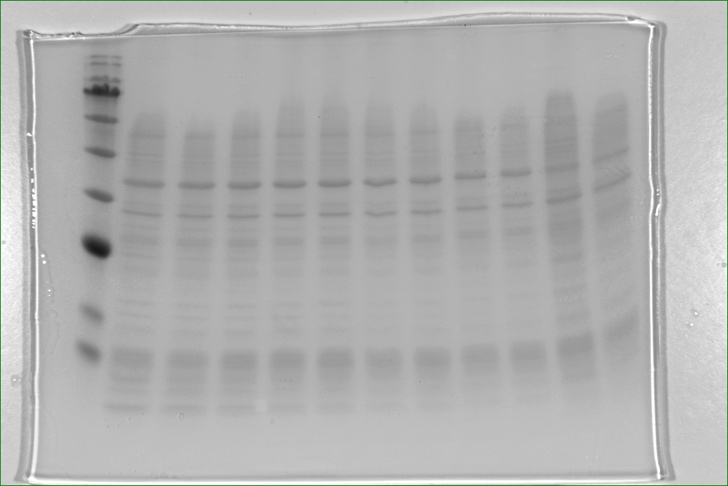


**Fraction No**


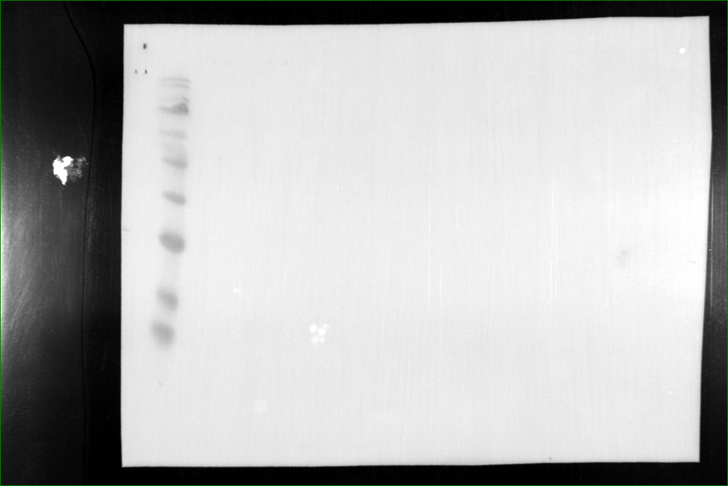

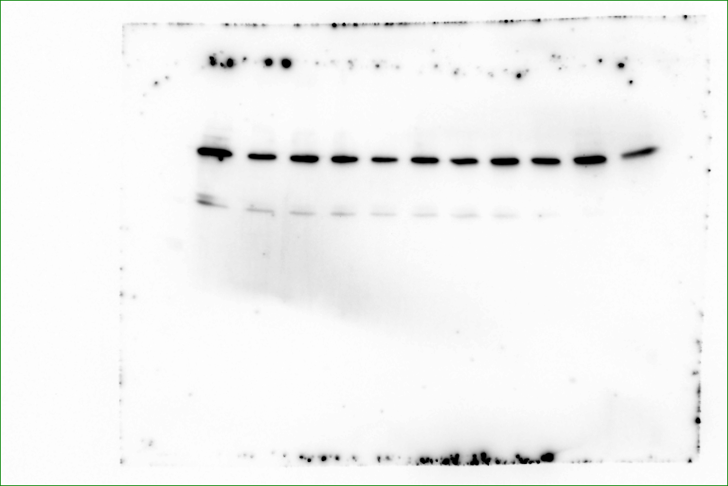


M

**Purification of hGPER protein using Ni-NTA affinity chromatography.**

**Full uncropped gel and blot image(s) for S2 Fig. C**

**Fraction No**


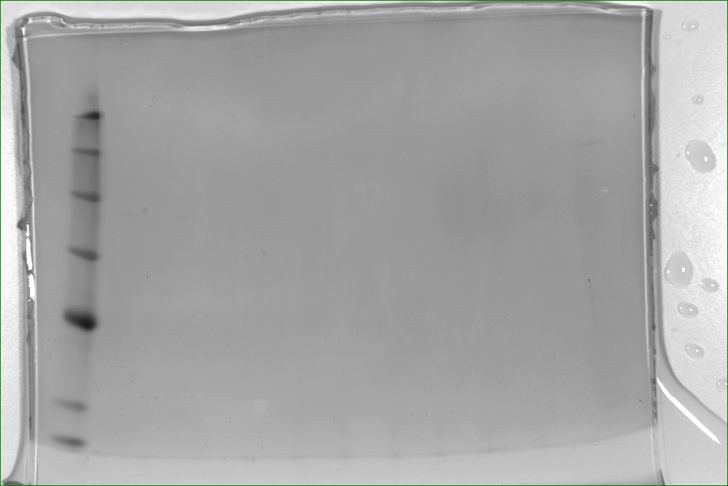


**Fraction No**

M


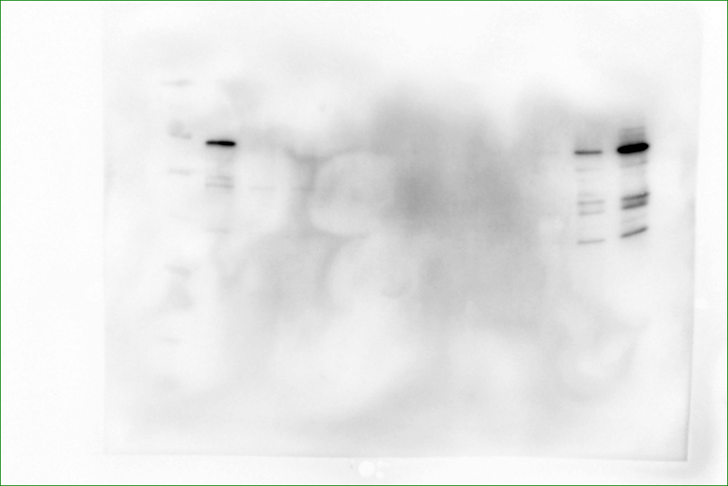

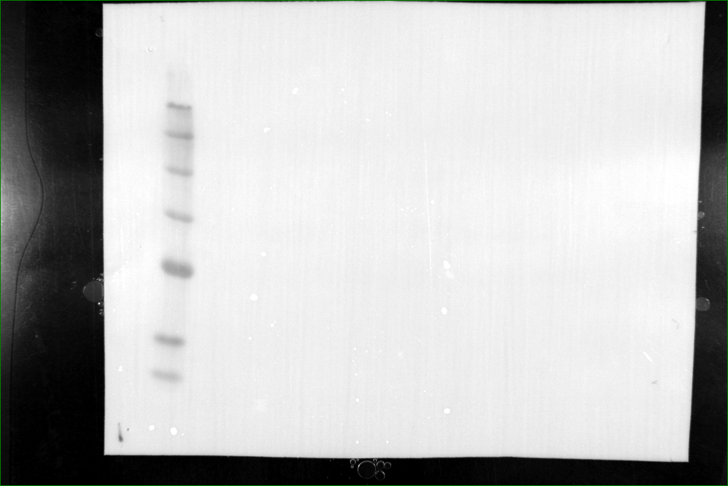


**Fraction No**


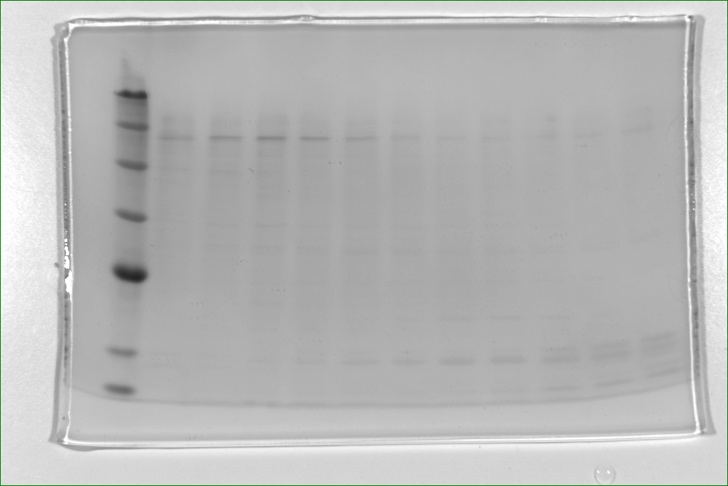

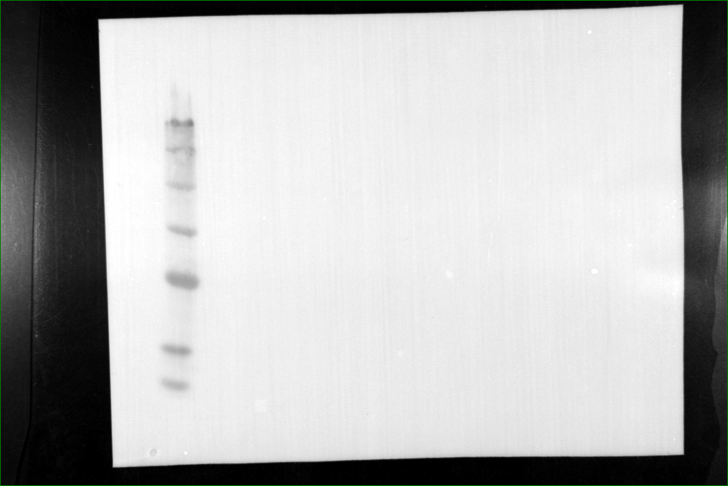


M

**Fraction No**

M

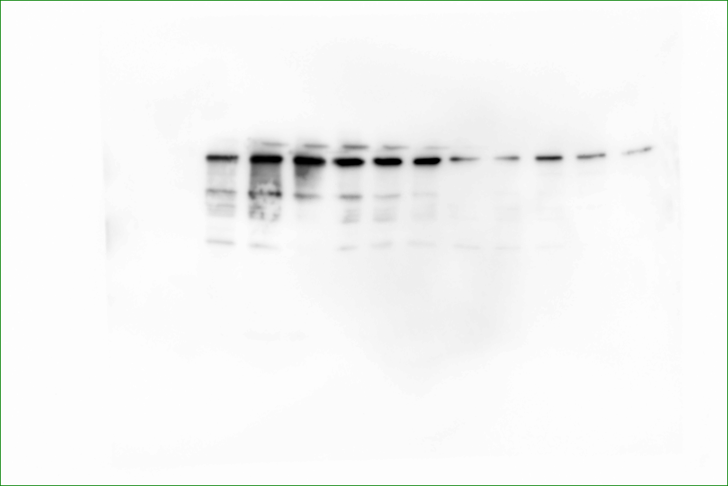


**Purification of hGPER protein using amino cellulose chromatography**.
